# Impact of Grazing on Soil Carbon and Microbial Biomass in Typical Steppe and Desert Steppe of Inner Mongolia

**DOI:** 10.1371/journal.pone.0036434

**Published:** 2012-05-04

**Authors:** Nan Liu, Yingjun Zhang, Shujuan Chang, Haiming Kan, Lijun Lin

**Affiliations:** Institute of Grassland Science, College of Animal Science and Technology, China Agricultural University, Beijing, People's Republic of China; Institut de Pharmacologie et de Biologie Structurale, France

## Abstract

The potential of grazing lands to sequester carbon must be understood to develop effective soil conservation measures and sustain livestock production. Our objective was to evaluate the effects of grazing on soil organic carbon (SOC), total nitrogen (TN), microbial biomass carbon (MBC) in Typical steppe and Desert steppe ecosystems, which are both important grassland resources for animal grazing and ecological conservation in China, and to derive region-specific soil C changes associated with different stocking rates (ungrazed, UG; lightly grazed, LG; moderately grazed, MG; heavily grazed, HG). This study substantiated that significant higher SOC, TN and MBC appeared with the treatment of LG in typical steppe. From 2004 to 2010, grazing treatments increased soil carbon storage in desert steppe, which was partly due to the grazing history. The higher MBC concentration and MBC/SOC suggest a great potential for carbon sequestration in the desert steppe ecosystem. The greater MBC in desert steppe than typical steppe was mainly the result of higher precipitation and temperature, instead of soil substrate. The change of MBC and the strong positive relationships between MBC and SOC indicated that MBC in the soil was a sensitive index to indicate the dynamics of soil organic carbon in both steppes in Inner Mongolia of China.

## Introduction

Carbon sequestration in rangeland ecosystems has emerged as an important service to sequester greenhouse gases and mitigate climate change. Grazing, as the most geographically expansive land use, occurs over a third of the earth's land surface and may potentially influence the storage of 10^9^ Mg year^−1^ of greenhouse gases as soil C [1]. In recent years, extensive work has been conducted toward improving understanding of C reserves of grazing lands and quantifying pools and fluxes. Grasslands store considerably more carbon in soil than in the vegetation [2]. Studies have shown that grazing can often promote C storage [3], [4]. Thus, understanding the change of soil C storage under grazing intensity is important to reduce greenhouse-gas emissions and mitigate climate change.

Grazing can have a direct impact on plant production and thereby on soil C inputs, and has been extensively studied [5]–[7]. The change of vegetation composition has proved to be an important factor in influencing soil carbon sequestration in grazing ecosystems [8]. Vegetation that has changed from a C_3_ dominated, to a more C_4_ dominated plant community due to grazing, can lead to SOC accumulating closer to the soil surface, making it more vulnerable to being lost to the atmosphere [9]. However, it is also reported that an increase in communities of C_4_ grasses which are tolerant of grazing and have more dense root systems and higher root-to-shoot ratios, at heavy grazing would result in increases in soil C and N [10], [11]. Grazing also influences the amount and composition of soil organic matter (SOM) [3], [12] through its effects on litter accumulation and decomposition [13], [14]. Soil microorganisms play a central role in decomposition and respiration, and influence C storage in soil. Soil microbial biomass, the living part of soil organic matter, functions as a transient nutrient sink and is responsible for decomposition and transformation of organic materials which are mostly derived from above and below-ground plant residues, and releasing nutrient from organic matter which is used by plants [15], [16]. Small changes in soil organic carbon in the short term are difficult to monitor, because of large background C concentrations and the natural variability of soils [17]. Microbial biomass carbon (MBC) generally comprises 1–4% of soil organic matter [18] and is the most active component of soil organic carbon that regulates biogeochemical processes in terrestrial ecosystems [19]. Soil MBC, as an important indicator of changes of soil quality and management practices [20], [21], is very sensitive to environmental changes [22]. Microbial biomass also acts as a small but labile reservoir of nutrients that contributes to maintaining long-term soil sustainability. In grazing lands, organic input from vegetation and excreta of animal can contribute to increased soil organic matter content and consequently cause an impact on soil biological processes. Thus, soil microbial biomass plays a critical role in grazing ecosystems as there is a large input of organic residue.

A dearth of information exists for the potential of C storage due to different grazing intensities in desert steppe ecosystems of Inner Mongolia [23], [24]. They generally reported that grazing-induced changes in litter input and erosion could influence soil organic matter content. However, microbial biomass has a close relationship with litter decomposition, which was not mentioned. Therefore, to better understand C cycling, it is important to gain an understanding of the MBC content that is affected by grazing and the relationship between SOC and MBC. Furthermore, we conducted the same research in the typical steppe. Of the 7.88 Mha of native rangeland in Inner Mongolia, desert steppe and typical steppe occupy 39% and 34%, respectively [25], and are thus two of the most important ecosystem types in Inner Mongolia. The specific aims of this study were to: (1) statistically investigate the effects of grazing pressure on SOC, TN, MBC, C/N, and MBC/SOC in the two different steppe ecosystems; (2) and to evaluate possible relationships of between SOC, TN, MBC and C/N.

## Methods

### Study Area

**Figure 1:**
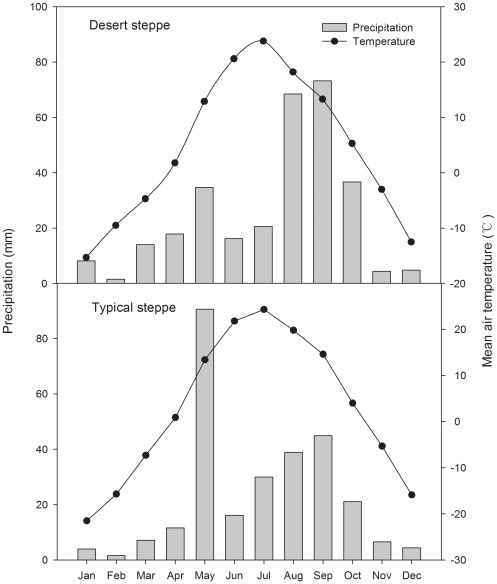
Mean monthly air temperature and rainfall distribution for typical steppe and desert steppe in 2010. Variation of annual average temperature and annual average precipitation from 1990–2010 in typical steppe and desert steppe.

The desert steppe is located at Siziwang Banner near Hohhot (41°47′N, 111°53′E; elevation: 1450 m) in western Inner Mongolia. This region is dry and windy in spring and hot in summer, with a mean annual precipitation of 280 mm and mean annual temperature of 3.4°C [24]. In 2010, higher precipitation appeared in August, and September, and higher temperatures appeared in July (Fig. 1). The soil is Kastanozem (FAO soil classification) with a loamy sand texture [23]. The average SOC, total N, P and K were 14.87 g/kg, 0.14%, 0.057% and 2.34%, respectively (Table 1). The dominant species were *Stipa breviflora* Griseb., *Artemisia frigida* Willd. and *Cleistogenes songorica* (Roshev.) Ohwi (C_4_ plant); associated species were *Convolvulus ammannii* Desr., *Heteropappus altaicus* (Willd.) Novopokr., *Neopallasia petinata* (Pall.) Poljak., *Kochia prostrata* (L.) Schrad.(C_4_ plant), *Caragana stenophylla* Pojark., and *Leymus chinensis* (Trin.) Tzvel.

**Table 1:** Mean values of soil characteristics of upper 30 cm.

site	pH	SD(g m^−3^)	SOC (g/kg)	TN (%)	TP(%)	TK (%)
Typical steppe	7.68	1.24	15.67	0.15	0.061	2.29
Desert steppe	7.45	1.26	14.87	0.14	0.057	2.34

SD, soil density; TP, total P; TK, total K.

The typical steppe is located in the Xilin River catchment on the Mongolian plateau near the Grassland Ecosystem Research Station of the Chinese Academy of Sciences (GERS,CAS) (43°38′N, 116°42′E; elevation: 1200 m) in Inner Mongolia. This area is characterized by a continental, semi-arid climate, with mean annual precipitation rate of 335 mm and a mean annual temperature of 0.7°C [26]. Typically, most precipitation falls within the growing season, thus favoring the productivity of steppe [27]. In 2010, higher precipitation appeared in May, August, and September, and the highest temperature appeared in July (Fig. 1). The predominant soil types of this area are Calcic Chestnuts and Calcic Chernozems with a fine-sand loess texture [28]. The average SOC, total N, P and K were 15.67 g/kg, 0.15%, 0.061% and 2.29%, respectively (Table 1). Dominant species in the typical steppe ecosystem were the perennial rhizomatous grass, *Leymus chinensis*, and the perennial bunchgrass, *Stipa grandis* [29], [30]; main associated species were *Cleistogenes squarrosa* (C_4_ plant), *Carex korshinsky*, and *Agropyron cristatum*.

### Experimental Design and Soil Sampling

In the desert steppe, before 1988, nomadic herders utilized the experimental grassland at a relatively light grazing intensity. Grassland degradation occurred as sheep numbers increased. Four different stocking rates 0, 0.91, 1.82, and 2.71 sheep/ha were compared in a randomized block design with three replicate blocks, which was established in 2004. These were classified as ungrazed (UG), lightly grazed (LG), moderately grazed (MG) and heavily grazed (HG). The utilized above ground biomass was 13, 26 and 39% for LG, MG and HG, respectively [31]. Ground cover was 19, 17, 15, and 13% in 2004, and 23, 17, 10, and 11% in 2008 for UG, LG, MG, and HG, respectively [32]. The four treatments were sampled for soil C in 2004 [32]. These sites were resampled in 2010 and these are the results presented. Plot size was approximately 4.4 ha. Castrated male sheep, two or three years old and averaging 39 kg live weight, were grazed for six months from the beginning of June to the end of November. Sheep were grazed from 6:00 am to 6:00 pm, and then penned at night.

In typical steppe, the experimental area had been moderately grazed by sheep until 2003 and afterwards, the area was rested for 2 years before being fenced for initiation of grazing treatments in June 2005. Four different stocking rates of ungrazed (UG), lightly grazed (LG), moderately grazed (MG), and heavily grazed (HG) for 0, 1.5, 4.5, and 9.0 sheep/ha, respectively, with two blocks were compared. The grazing intensity was defined by herbage allowance. Average seasonal herbage allowance target ranges on grazing plots of <1.5, 3–4.5, >12 kg dry matter per kg live weight were aimed to describe LG, MG and HG, respectively [26]. Compared with UG, remnant vegetation at the end of the grazing season (September) was 109, 38, and 15% in 2005, and 96, 25, and 18% in 2006 for LG, MG and HG, respectively [28]. The grazing animals were 15-month-old female sheep, averaging 35 kg live weight, and were kept continuously on the plots day and night throughout the grazing season. Except for LG, the standard plot size was 2 ha. In order to achieve a minimum of 6 sheep per plot, the LG plot was 4 ha. Grazing lasted from the beginning of June to the beginning of September and coincided with the growing season.

### Sampling Procedures

The soil was sampled along five 50 m transects within each zone of grazing intensity in mid-August of 2010. Five soil cores were collected manually with a soil sampler, at 10 m intervals along each 50 m sampling transect. The soil sampler was a metal cylinder (cylinder: diameter, 5 cm; length, 20 cm, the total length of the sampler was 1.3 m), and was vertically inserted into the soil for sampling. At each sampling point, coring was carried out at four depths (0–5 cm, 5–10 cm, 10–20 cm and 20–30 cm). Sampling in the upper layer (0–5 cm) was most likely to maximize the chances of detecting grazing effects, because this layer contained the highest amount of both soil C and N, especially the ratio of active fraction (MBC) to overall-SOM [33], [34]. In 2004, the soil sampling procedure was similar to 2010, but there were 9 rather than 25 soil cores. Soil bulk density was also assessed on separate soil cores (five cores per plot) from each site (100 cm^3^–volume) obtained from the four layers. After removing roots and stones by sieving with 2 mm mesh, soil samples were divided into two parts, and one was directly sealed in ziplock bags and put into coolers. They were transported to the soil storage facility of China Agriculture University, stored in refrigerated conditions (4°C), and then analyzed for Microbial Biomass Carbon. All remaining samples were measured the gravimetrical water content (oven-dried, 105°C, 12 h), soil organic carbon and total nitrogen (air-dried).

### Laboratory Analysis

The SOC concentration was estimated using an autoanalyzer (TOC-VCPN, SSM-5000A, Shimadzu, Japan). The inorganic C was removed from soil sampling with 1 M HCl prior to SOC determination, so the total C concentration was equal to the organic carbon concentration. The TN concentration was determined using the modified Kjeldahl wet digestion procedure [35], using a 2300 Kjeltec Analyzer Unit (FOSS, Sweden). We calculated the mass of SOC and the mass of soil TN on a ground area basis up to a 30-cm depth as follows:
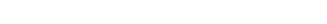


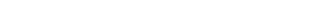
where SD_i_, BD_i_, OC_i_, and TN_i_ represent the soil depth (cm), bulk density (g cm^−3^), organic C concentration (g/kg), and total N concentration (g/kg) of the ith layer, respectively; i = 1, 2, 3, and 4.

MBC was determined using the chloroform fumigation method [36], [37]. Organic (microbial) carbon concentration in each sample (control and chloroform-fumigated) was determined using the autoanalyzer described above. The difference between the carbon in the controls and chloroform-fumigated samples was used to calculate the MBC with the following equation:
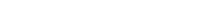
where *K*_ec_ is the correction factor related to the proportion of microbial biomass or the coefficient of extracting microbial carbon from the soil, *C*_control_ is the microbial biomass carbon from the control (unfumigated) samples, and *C*_fumigate_ is the microbial biomass carbon from the fumigated samples. *K*_ec_ value is 2.64 [34].

### Data and Statistical Analysis

To evaluate grazing effect on SOC, TN, and C∶N ratio, a general linear model (GLM) was employed for analysis of variance (ANOVA) among stocking rates and depths within a site. The effect of grazing intensity on MBC was tested using analysis of covariance, with a covariate of soil water content. The LSD test was used for all mean comparisons. A confidence interval of 95% (α = 0.05 level of significance) was used for analysis of significant difference, unless otherwise stated. A simple linear regression analysis was used to determine the relationship between MBC and soil moisture. The Pearson Correlation was used to investigate the relationships between MBC, SOC, TN and C∶N ratio. All statistical analyses were performed using the software program SPSS, ver. 16.0 (SPSS Inc., Chicago, IL, USA).

## Results

**Figure 2:**
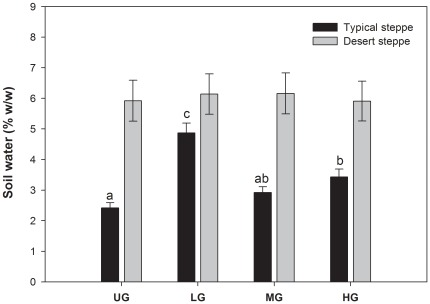
Mean soil water content (%, w/w) in two steppes for four treatments. The error bars indicate the standard error. Means sharing the same letters were not significantly different (p<0.05). UG, ungrazed; LG, lightly grazed; MG, moderately grazed; HG, heavily grazed.

In the sampling year of 2010, the monthly rainfall distribution for desert steppe was slightly higher than that for typical steppe, especially in August and September (Fig. 1). Grazing intensity significantly affected soil water in typical steppe (*P*<0.05), but not in desert steppe. A relatively higher soil water content was found in the desert steppe compared to the typical steppe (Fig. 2).

### Soil Organic Carbon and Total Nitrogen

Grazing intensity was found to significantly affect SOC, TN and C/N in typical steppe (*P*<0.05), and but not in desert steppe. Depth was a significant factor for all response variables in both steppes except for C/N. Grazing intensity and depth interactions were not significant for any response in both steppes (Table 2).

**Table 2:** Partial ANOVA table showing degrees of freedom (DF) and *P*-values from GLM analysis (at α = 0.05) of TN, SOC, MBC and C∶N ratio in the study sites across grazing intensity (GI) and depths (D).

Effect	DF	TN	SOC	C∶N
Desert steppe				
GI	3	0.455NS	0.542 NS	0.841 NS
D	3	<0.001	<0.001	0.631NS
GI×D	9	0.561NS	0.912NS	0.912NS
Typical steppe				
GI	3	<0.001	<0.001	0.003
D	3	<0.001	<0.001	0.613NS
GI×D	9	0.955NS	0.747NS	0.999NS

NS not significant.

In typical steppe, significantly higher SOC and TN in the LG treatment was found compared to other treatments at all depths, and were in the order of LG>HG>MG>UG (Table 3). SOC and TN concentration decreased significantly with depth, but there were no differences across treatments for a given depth in desert steppe. The mass of SOC for 0–30 cm soil depth was in the order of UG>LG>HG>MG, and higher TN was present in LG and HG treatments in the desert steppe (Table 3). Values of C/N were lower in ungrazed than grazed treatments in typical steppe, but were not different in desert steppe (Table 4).

**Table 3:** Concentration and mass of soil organic carbon (SOC) and total nitrogen (TN) for soils from ungrazed (UG), lightly grazed (LG), moderately grazed (MG), and heavily grazed (HG) sites sampled in 2010 in the Desert and Typical steppe, Inner Mongolia.

	SOC					TN				
	Concentration (g kg^−1^)				Mass (Mg ha^−1^)	Concentration (g kg^−1^)				Mass (Mg ha^−1^)
Soil depth(cm)	0–5	5–10	10–20	20–30	0–30	0–5	5–10	10–20	20–30	0–30
Desert steppe										
UG	15.09	14.54	14.22	13.84	53.79	1.61	1.64	1.52	1.42	5.74
LG	14.62	14.75	13.23	11.54	48.29	1.66	1.63	1.56	1.40	5.83
MG	13.69	13.94	13.11	11.52	47.27	1.64	1.71	1.48	1.34	5.57
HG	14.27	14.17	12.71	11.21	47.97	1.78	1.66	1.57	1.39	6.01
s.e.	0.28	0.34	0.42	0.57	1.16	0.04	0.02	0.02	0.02	0.11
Typical steppe										
UG	15.33a	13.18a	11.13a	8.61a	45.64a	1.85a	1.59a	1.45a	1.12a	5.78
LG	23.66c	18.56c	16.84c	14.25b	55.37c	2.46c	2.06b	1.89b	1.57c	6.11
MG	19.15b	14.90ab	13.54b	10.49a	47.99ab	2.12b	1.66a	1.54a	1.24ab	5.46
HG	20.24b	16.52bc	15.42bc	13.77b	53.11bc	2.11b	1.81ab	1.67ab	1.47bc	5.81
s.e.	0.71	0.49	0.54	0.57	1.30	0.05	0.06	0.05	0.06	0.14

Different small letters indicate significant differences between different stocking rates (*P*<0.05).

**Table 4:** Mean (SE) C∶N ratios (incorporating all depths) and mean (SE) MBC∶SOC ratios (incorporating 0–5 cm and 5–10 cm depths) with different grazing pressure for two sites.

Treatment	UG	LG	MG	HG
C/N				
Typical Steppe	8.08(0.24)a	9.24(0.19)b	8.96(0.30)b	9.43(0.25)b
Desert steppe	8.801(0.21)a	8.71(0.26)a	8.54(0.21)a	8.54(0.30)a
MBC/SOC (mg MBC g^−1^SOC)				
Typical Steppe	14.08(1.82)a	17.62(1.22)a	17.66(1.50)a	14.13(1.81)a
Desert steppe	27.34 (1.71)a	26.37 (1.59)a	28.93(1.86)a	27.41 (2.13)a

Data in different letters means significant differences between different stocking rates (*P*<0.05).

In the desert steppe, 6 years after initiation of the grazing treatments in 2004, the concentration of SOC has increased in all depths and treatments except for UG treatment (0–10 cm). These observed differences between the 2004 and 2010 sampling resulted in increases (0–10 cm: 3.2% in LG, 1.1% in MG, and 2.4% in HG, separately; 10–20 cm: 5.8% in UG, 16.7% in LG, 10.4% in MG, and 6.6% in HG, separately) of SOC with grazing treatments (Table 5).

**Table 5:** Comparison of concentration of SOC in desert steppe between 2004 and 2010.

SOC(g/k)	2004†		2010	
	0–10 cm	10–20 cm	0–10 cm	10–20 cm
UG	15.56±0.73	13.44±0.63	14.82±0.63(−4.8)‡	14.22±1.41(+5.8)
LG	14.24±0.73	11.34±0.93	14.69±0.67(+3.2)	13.23±0.67(+16.7)
MG	13.67±1.41	11.88±0.38	13.82±0.45(+1.1)	13.11±0.51(+10.4)
HG	13.89±1.41	11.92±0.52	14.22±0.54(+2.4)	12.71±0.55(+6.6)

† from Wang (2009).

‡ Figures in parentheses indicate the percent change between the 2004 and 2010 sampling dates.

### Microbial Biomass Carbon Concentration

**Figure 3:**
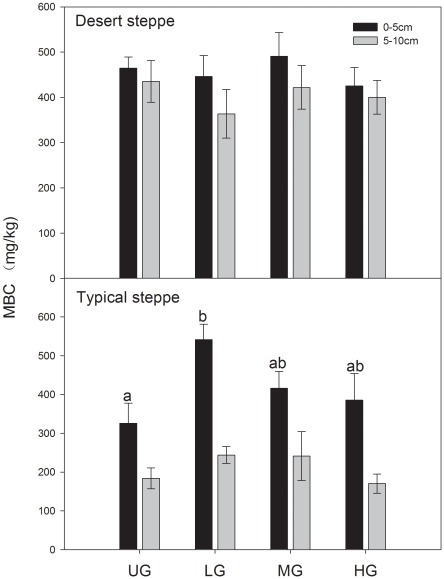
Mean MBC concentration in two types of grassland, inclusive of two soil depths and four stocking rates. The error bars indicate the standard error. Means sharing the same letters were not significantly different (p<0.05). UG, ungrazed; LG, lightly grazed; MG, moderately grazed; HG, heavily grazed.

In typical steppe, MBC concentrations were far higher in the surface soil (0–5 cm) than in the sub-layer soil. In addition, MBC content was significantly higher in LG than in UG treatments in the 0–5 cm layer (Fig. 3). There were no significant differences due to grazing intensity in MBC concentration in desert steppe, and the MBC concentrations were only slightly higher in the surface soil (0–5 cm) than in the sub-layer soil (Fig. 3). Soil MBC in both steppes were positively correlated with soil water (R = 0.554, *P*<0.05 in typical steppe; R = 0.382, *P*<0.05 in desert steppe, respectively).

### Relationships between SOC, TN, MBC and C/N

The MBC generally comprised 1–3% of SOC, with the proportion being consistently greater in desert steppe than in typical steppe. In the two steppes, there were no significant differences across grazing treatments, and the higher MBC/SOC values appeared in the MG treatment (Table 4). Correlations between SOC, TN, and MBC were significantly positive and strong (*P*<0.001) at both sites. At both study areas, SOC and C/N exhibited extremely strong positive correlations (*P*<0.001 in typical steppe and desert steppe). However, TN and C/N also exhibited extremely strong negative correlations (*P*<0.001) in desert steppe (Table 6).

**Table 6:** Correlation between SOC, TN, MBC, and C/N for study sites (incorporating all depths and all stocking rates, n = 240 in desert steppe, n = 160 in typical steppe).

Site	Parameter	TN	MBC	C/N
Typical Steppe	SOC	0.798† **	0.697**	0.478**
	TN		0.566**	−0.128
	MBC			0.346*
Desert Steppe	SOC	0.645**	0.784**	0.388**
	TN		0.613**	−0.444**
	MBC			0.137

** means significant difference at the *P*≤0.001 level;

* means significant difference at the *P*≤0.05 level;

† All values are Pearson correlation coefficient (range 0–1).

## Discussion

### Difference in C and N due to Grazing Intensity

Proper management of rangelands, or restoration of degraded rangelands through improved management, can sustain or improve soil C sequestration and contribute to mitigation of atmospheric CO_2_ increase [38]–[40]. However, studies conducted on Inner Mongolia pastures showed variable responses for carbon storage with grazing practices. Soil C levels are influenced by above-ground biomass and productivity of vegetation due to environmental, ground litter accumulation and decomposition, below-ground root mass and distribution, physical and biological conditions in the soil and the history of grassland utilization [23], [25], [41]–[43].

In the desert steppe, the increase of C levels in all treatments was partly due to the grazing history. The grassland has been over-grazed since 1988 because of an increase in population in the area. Prior to 2004, when our experiment started, the grassland was seriously degraded. Thus, the stocking rates we studied may have been lower than the actual stocking rates used prior to the experiment, resulting in an increase of C in this period. The maximum level of SOC appeared to be related to a particular level of grazing pressure, such that some grazing pressure was required to maximise SOC sequestration, but an excessive grazing pressure could reduce the SOC. In the typical steppe, significantly higher SOC in LG treatment compared to other treatments was consistent with the findings of other research [11]. Unfortunately, we did not measure C in the plots prior to applying the grazing treatments, but the experimental design should partly make up for the failure to take initial measurements. These results suggested that LG treatments in grassland treatment were essential for increases in soil carbon stores, and these were also proved by the increase of soil carbon from 2004 to 2010, as the highest increase appeared in LG treatment of desert steppe. In the previous research, higher litter accumulation appeared in the LG treatment relative to MG and HG treatments in the typical steppe [26] and the adjacent desert steppe [23]. This resulted in higher potential C inputs from both aboveground litter production and belowground root mass [10]. Animal traffic enhances physical breakdown and incorporation of litter into the soil [13], which can increase the rate of decomposition of litter and transfer of C and nutrients into the soil [14]. These responses are likely to happen in grazing treatments that maintained a higher carbon input from root, litter and excreta while an ungrazed treatment would strongly decrease this input and promote aboveground allocation. Additionally, it was reported lower total soil C and N concentrations due to reduced plant inputs following exclusion of grazing [6]. This may result in higher carbon stores in grazing lands than the lands where grazing was excluded in typical steppe and the decrease in UG treatment from 2004 to 2010 in desert steppe. Additionally, grazing could promote plant below-ground allocation and root exudation of carbon [44]. In LG of the desert steppe, the accumulated litter had a higher C/N ratio than soil [24] and was incorporated into soil to increase soil C/N ratio. C∶N ratios increased under grazing conditions, and the highest value appeared in the HG treatment, which suggests potential N limitations for SOM formation and SOC accumulation [45] under heavy grazing.

From 2004 to 2010, an increase of soil carbon concentration occurred in UG, LG, MG and even HG treatments in desert steppe. Additionally, SOC and TN with HG appeared to be higher than with MG in both typical and desert steppes. All these responses reflect that short-term heavy grazing has a better potential for soil C and N sequestration [46], [47]. Though increased forage consumption in the HG treatment resulted in a loss of above-ground plant N, it is estimated that 80 to 95% of consumed plant N is returned in the form of excreta [48]. The typical steppe site had sheep retained on plots overnight, ensuring a greater amount of nutrient was returned to the soil than the desert steppe and may help to show the higher C and N in soil. Much research has shown that the increase of C_4_ grasses with dense shallow root systems was probably a major contributor to higher C with heavy grazing. Previous works have also found the same result for the increase of C_4_ plant in HG of the same plots in typical and desert steppe [26], [32], [49]. This may result in the higher C in 0–5 cm and 5–10 cm soil depth of HG treatment compared to MG treatment, but not in 10–20 cm and 20–30 cm soil depth. Consequently, it is a likely reason to explain the higher C in heavy grazing lands but requires further research in steppe in China.

### Changes in Microbial Biomass Carbon

Soil microbial biomass responds rapidly to land management strategy changes and is an indicator of soil health in the carbon cycle [22]. In the desert steppe, the similar soil organic matter content among grazing treatments was responsible for unobvious differences in MBC, while multiple factors were responsible for the significant differences in MBC among the treatments in the typical steppe. A reduction of MBC concentration occurred with the increase of grazing intensity, and this finding was similar to those of many other studies [20], [21], [50]. Higher organic matter inputs from plant litter and root exudates may have enhanced the rate of MBC production in the soil [51]. This response would be most likely to happen in the LG treatment that maintained higher MBC concentration than other treatments. Furthermore, litter accumulation in the surface soil where roots dominate indicates a greater opportunity for nutrient availability and cycling, which maintained MBC in a higher level in the topsoil layer (0–5 cm) relative to the sub-layer (5–10 cm) [14]. In concert with the change of SOC, higher MBC content in grazed soils indicated a higher potential opportunity for nutrient availability and carbon cycling relative to ungrazed soil. Microbial biomass reflects soil assimilative and mineralization capacity. In previous research, the potential N mineralization in the same field was higher in LG and MG treatments relative to HG and UG treatments, and the lowest value appeared in UG treatment [52]. This result is consistent with the change of MBC. In addition, heavy grazing, especially trampling, destroys the soil environment (e.g. increases bulk density, and decreases soil porosity and aggregation), and then disturbs the growth and metabolization of microorganisms [53], resulting in the lowest MBC concentration.

The soil water content is an important factor to consider as it is directly related to the soil microbial activity [54]. Greater soil moisture has contributed to greater MBC content in desert steppe. Grazing intensity lead to the variation in MBC as a direct result of the soil water content in typical steppe, while the unobvious differences in MBC likely due to the similar water content among grazing treatments in desert steppe. Other studies are in agreement with the data presented, with higher MBC as soil water content increases [55], [56]. Soil water availability can also indirectly influence soil microorganisms via increasing decomposition of litter and SOM and consequent labile C substrates [55]. Greater MBC as a result of higher soil water and litter in LG treatment of typical steppe, supports the above argument. The increase of root productivity and soil temperature are also reasons leading to greater MBC [57]. Although it is not possible to directly compare results between the typical steppe and the desert steppe due to differences in grazing treatments and inherent differences in environment and soil conditions, relative to a higher quality substrate of soil in typical steppe (Table 1), we speculate that the higher MBC content in desert steppe relative to the typical steppe occurred largely as a result of higher precipitation and temperature. The higher MBC content indicated a greater carbon turnover and greater abundance of metabolizable C in desert steppe [58].

### The Relationship of MBC with SOC

Soil MBC/SOC ratio is an index of the accumulation potential of microbial biomass carbon relative to the organic carbon [17]. It has been proposed for evaluating grazing effects due to the fact that it is a sensitive measure of soil health, and considered superior to its single components (MBC or SOC) and to other parameters, since it represents a combination of microbial activity and key soil resources [17], [18], [59]. In the desert steppe, the higher soil moisture resulted in higher MBC content and thereby higher efficiency in the conversion of SOC into MBC. This was proved by the higher MBC∶SOC ratio in the desert steppe relative to the typical steppe in this study.

Apart from the close relationship between soil N and C, the correlation between MBC and SOC was positive and strong in both steppes, showing that content of MBC in the soil was a sensitive index to indicate the dynamics of soil organic carbon in the growing seasons of both steppes. Our results generally agree with those reported in other studies, which described similar relationships [34], [60].

### Conclusions

Light grazing treatment could be construed as proper management to sustain soil C sequestration in desert and typical steppe. Although an increase of soil C and N was estimated in HG treatment in the two steppes, it is estimated that a decrease of MBC appeared with the increase of grazing intensity. This result combined with the strong and positive relationship between MBC and SOC appearing in the two steppes indicates that MBC was a sensitive index to indicate the dynamics of soil organic carbon. The higher MBC concentration and MBC∶SOC ratio in the desert steppe indicates that MBC recovered more rapidly than the SOC in the condition of favorable precipitation and temperature, but it is still need confirm the importance of the MBC/SOC ratio in soil carbon dynamics in future.

## References

[1] Scurlock JM, Hall DO (1998). The global carbon sink: a grassland perspective.. Global Change Biology.

[2] White R, Murray S, Rohweder M (2000). Pilot analysis of global ecosystems: Grassland ecosystems.

[3] Frank AB, Tanaka DL, Hofmann L, Follett RF (1995). Soil carbon and nitrogen of Northern Great Plains grasslands as influenced by long-term grazing.. Journal of Range Management.

[4] Reeder JD, Schuman GE (2002). Influence of livestock grazing on C sequestration in semi-arid mixed-grass and short-grass rangelands.. Environmental Pollution.

[5] Steffens M, Kölbl A, Totsche KU, Kogel-Knabner I (2008). Grazing effects on soil chemical and physical properties in a semiarid steppe of Inner Mongolia (P.R. China).. Geoderma.

[6] Kelly RH, Burke IC, Lauenroth WK (1996). Soil organic matter and nutrient availability responses to reduced plant inputs in shortgrass steppe.. Ecology.

[7] Lü FM, Lü XT, Liu W, Han X, Zhang GM (2011). Carbon and nitrogen storage in plant and soil as related to nitrogen and water amendment in a temperate steppe of northern China.. Biology and Fertility of Soils.

[8] Bagchi S, Ritchie ME (2010). Introduced grazers can restrict potential soil carbon sequestration through impacts on plant community composition.. Ecology Letters.

[9] Ingram LJ, Stahl PD, Schuman GE, Buyer JS, Vance GF (2008). Grazing impacts on soil carbon and microbial communities in a mixed-grass ecosystem.. Soil Science Society of American Journal.

[10] Reeder JD, Schuman GE, Morgan JA, Lecain DR (2004). Response of organic and inorganic carbon and nitrogen to long-term grazing of the shortgrass steppe.. Environment Management.

[11] Schuman GE, Reeder JD, Manley JT, Hart RH, Manley WA (1999). Impact of grazing management on the carbon and nitrogen balance of a mixed-grass rangeland.. Ecological Applications.

[12] Dormaar JF, Willms WD (1990). Effect of grazing and cultivation on some chemical properties of soils in the mixed prairie.. Journal of Range Management.

[13] Naeth MA, Bailey AW, Pluth DJ, Chanasyk DS, Hardin RT (1991). Grazing impacts on litter and soil organic matter in mixed prairie and fescue grassland ecosystems of Alberta.. Journal of Arid Environments.

[14] Shariff AR, Biondini ME, Grygiel CE (1994). Grazing intensity effects on litter decomposition and soil nitrogen mineralization.. Journal of Range Management.

[15] Smith L, Paul EA, Bollag JM, Stotzky G (1990). The significance of soil microbial biomass estimations.. Soil Biochemistry.

[16] Ananyeva ND, Demkina TS, Jones WJ, Cabrera ML, Steen WC (1999). Microbial biomass in soils of Russia under long term management practices.. Biology and Fertility of Soils.

[17] Sparling GP (1992). Ratio of microbial biomass carbon to soil organic carbon as a sensitive indicator of changes in soil organic matter.. Australian Journal of Soil Research.

[18] Anderson TH, Domsch KH (1989). Ratios of microbial biomass carbon to total organic carbon in arable soils.. Soil Biology & Biochemistry.

[19] Paul EA, Clark FE (1996). Soil Microbiology and Biochemistry.

[20] Holt JA (1997). Grazing pressure and soil carbon, microbial biomass and enzyme activities in semi-arid northeastern Australia.. Applied Soil Ecology.

[21] Wang C, Long R (2008). Changes in soil organic carbon and microbial biomass carbon at different degradation successional stages of alpine meadows in the headwater region of three rivers in China.. Journal of Applied & Environmental Biology.

[22] Nielsen NM, Winding A, Binnerup S (2002). Microorganisms as indicators of soil health. Ministry of the Environment.. National Environmental Research Institute.

[23] Li CL, Hao XY, Zhao ML, Han GD, Willms WD (2008). Influence of historic sheep grazing on vegetation and soil properties of a Desert Steppe in Inner Mongolia.. Agriculture Ecosystems and Environment.

[24] Lin Y, Hong M, Han GD, Zhao ML, Bai YF (2010). Grazing intensity affected spatial patterns of vegetation and soil fertility in a desert steppe.. Agriculture, Ecosystems and Environment.

[25] Han GD, Hao XY, Zhao ML, Wang MJ, Ellert BH (2008). Effect of grazing intensity on carbon and nitrogen in soil and vegetation in a meadow steppe in Inner Mongolia.. Agriculture Ecosystems and Environment.

[26] Schönbach P, Wan HW, Gierus M, Bai YF, Müller K (2011). Grassland responses to grazing: effects of grazing intensity and management system in an Inner Mongolian steppe ecosystem.. Plant and Soil.

[27] Yu M, Ellis JE, Epstein HE (2004). Regional analysis of climate, primary production, and livestock density in Inner Mongolia.. Journal of Environmental Quality.

[28] Schönbach P, Wan H, Schiborra A, Gierus M, Bai Y (2009). Short-term management and stocking rate effects of grazing sheep on herbage quality and productivity of Inner Mongolia steppe.. Crop & Pasture Science.

[29] Bai YF, Han XG, Wu JG, Chen ZZ, Li LH (2004). Ecosystem stability and compensatory effects in the Inner Mongolia grassland.. Nature.

[30] Xiao XM, Wang YF, Jiang S, Ojima DS, Bonham CD (1995). Interannual variation in the climate and above-ground biomass of Leymus chinense steppe and Stipa grandis steppe in the Xilin river basin, Inner Mongolia, China.. Journal of Arid Environment.

[31] Wang Z, Jiao SY, Han GD, Zhao ML, Willms WD (2011). Impact of Stocking Rate and Rainfall on Sheep Performance in a Desert Steppe.. Rangeland Ecology & Management.

[32] Wang ZW (2009). Effect of stocking rate on ecosystem stability of Stipa Breviflora desert steppe..

[33] Du Preez CC, Snyman WA (1993). Organic matter content of a soil in a semi-arid climate with three long-standing veld conditions.. African Journal of Range & Forage Science.

[34] Shrestha G, Stahl PD (2008). Carbon accumulation and storage in semi-arid sagebrush steppe: Effects of long-term grazing exclusion.. Agriculture ecosystems and environment.

[35] Gallaher RN, Weldon CO, Boswell FC (1976). A semi-automated procedure for total nitrogen in plant and soil samples.. Soil Science Society of American Journal.

[36] Vance ED, Brookes PC, Jenkinson DS (1987). An extraction method for measuring soil microbial biomass C.. Soil Biology & Biochemistry.

[37] Brookes PC, Landman A, Pruden G, Jenkinson DS (1985). Chloroform fumigation and the release of soil nitrogen: a rapid direct extraction method to measure microbial biomass nitrogen in soil.. Soil Biology & Biochemistry.

[38] Abril A, Bucher EH (2001). Overgrazing and soil carbon dynamics in the western Chaco of Argentina.. Applied Soil Ecology.

[39] Derner JD, Boutton TW, Briske DD (2006). Grazing and ecosystem carbon storage in the North American Great Plains.. Plant and Soil.

[40] Derner JD, Schuman GE (2007). Carbon sequestration and rangelands: a synthesis of land management and precipitation effects.. Journal of Soil and Water Conservation.

[41] Su YZ, Li YL, Cui JY, Zhao WZ (2005). Influences of continuous grazing and livestock exclusion on soil properties in a degraded sandy grassland, Inner Mongolia, northern China.. Catena.

[42] Su YZ, Zhao HL, Zhao TH (2003). Influences of grazing and exclosure on carbon sequestration in degraded sandy grassland, Inner Mongolia, north China.. Journal of Agricultural Research.

[43] He NP, Yu Q, Wu L, Wang YS, Han XG (2008). Carbon and nitrogen store and storage potential as affected by land-use in a *Leymus chinensis* grassland of northern China.. Soil Biology & Biochemistry.

[44] Hamilton EW, Frank DA (2001). Can plants stimulate soil microbes and their own nutrient supply? Evidence from a grazing tolerant grass.. Ecology.

[45] Pineiro G, Paruelo JM, Oesterbeld M, Jobbagy EG (2010). Pathways of grazing effects on soil organic carbon and nitrogen.. Rangeland Ecology & Management.

[46] Gao YH, Luo P, Wu N, Yi SL, Chen H (2007). Biomass and nitrogen responses to grazing intensity in an alpine meadow on the eastern Tibetan Plateau.. Journal of Ecology.

[47] Gao YH, Schumann M, Chen H, Wu N, Luo P (2009). Impacts of grazing intensity on soil carbon and nitrogen in an alpine meadow on the eastern Tibetan Plateau.. Journal of Food, Agriculture & Environment.

[48] Heady HF, Child RD (1994). Rangeland ecology and management..

[49] Yang HY, Bai F, Li YH, Han XG (2009). Response of plant species composition and community structure to long-term grazing in typical steppe of Inner Mongolia.. Chinese Journal of Plant Ecology.

[50] Northup BK, Brown JR, Holt JA (1999). Grazing impacts on the spatial distribution of soil microbial biomass around tussock grasses in a tropical grassland.. Applied Soil Ecology.

[51] Bird SB, Herrick JE, Wander MM, Wright SF (2002). Spatial heterogeneity of aggregate stability and soil carbon in semi-arid rangeland.. Environment Pollution.

[52] Yang Y (2010). Effect of grazing intensity on soil net nitrogen mineralization in typical steppe of Inner Mongolia..

[53] Wang QL, Wang CT, Du YG, Cao GM (2008). Grazing impact on soil microbial biomass carbon and relationships with soil environment in alpine Kobresia meadow.. Acta Prataculturae Sinica.

[54] Frazão LA, Piccolo MC, Feigl BJ, Cerri CC, Cerri CEP (2010). Inorganic nitrogen, microbial biomass and microbial activity of a sandy Brazilian Cerrado soil under different land uses.. Agriculture Ecosystems and Environment.

[55] Liu WX, Xu WH, Hong JP, Wan SQ (2010). Interannual variability of soil microbial biomass and respiration in responses to topography, annual burning and N addition in a semiarid temperate steppe.. Geoderma.

[56] Nardoto GB, Bustamante MMC (2003). Effects of fire on soil nitrogen dynamics and microbial biomass in savannas on Central Brazil.. Pesquisa Agropecuária Brasileira.

[57] Harris WN, Moretto AS, Distel RA, Bouttona TW, Boo RM (2007). Fire and grazing in grasslands of the Argentine Caldenal: Effects on plant and soil carbon and nitrogen.. Acta Oecologica.

[58] Kieft TL (1994). Grazing and plant-canopy effects on semiarid soil microbial biomass and respiration.. Biology and Fertility of Soils.

[59] Insam H, Domsch KH (1988). Relationship between soil organic carbon and soil microbial biomass on chronosequences of reclamation sites.. Microbial Ecology.

[60] Anderson JD, Ingram LJ, Stahl PD (2008). Influence of reclamation management practices on microbial biomass carbon and soil organic carbon accumulation in semiarid mined lands of Wyoming.. Applied Soil Ecology.

